# Lipid Antigen Presentation by CD1b and CD1d in Lysosomal Storage Disease Patients

**DOI:** 10.3389/fimmu.2019.01264

**Published:** 2019-06-04

**Authors:** Catia S. Pereira, Begoña Pérez-Cabezas, Helena Ribeiro, M. Luz Maia, M. Teresa Cardoso, Ana F. Dias, Olga Azevedo, M. Fatima Ferreira, Paula Garcia, Esmeralda Rodrigues, Paulo Castro-Chaves, Esmeralda Martins, Patricio Aguiar, Mercè Pineda, Yasmina Amraoui, Simona Fecarotta, Elisa Leão-Teles, Shenglou Deng, Paul B. Savage, M. Fatima Macedo

**Affiliations:** ^1^CAGE, Instituto de Biologia Molecular e Celular (IBMC), Universidade do Porto, Porto, Portugal; ^2^CAGE, Instituto de Investigação e Inovação em Saúde (i3S), Universidade do Porto, Porto, Portugal; ^3^Departamento de Química, Universidade de Aveiro, Aveiro, Portugal; ^4^UniLipe, Instituto de Biologia Molecular e Celular (IBMC), Universidade do Porto, Porto, Portugal; ^5^Centro de Referência de Doenças Hereditárias do Metabolismo (DHM), Centro Hospitalar de São João, Medicina Interna, Porto, Portugal; ^6^Centro de Referência de Doenças Lisossomais de Sobrecarga, Hospital da Senhora da Oliveira, Guimarães, Portugal; ^7^Centro de Referência de Doenças Hereditárias do Metabolismo (DHM), Hematologia Clínica, Centro Hospitalar de São João, Porto, Portugal; ^8^Centro de Referência de Doenças Hereditárias do Metabolismo (DHM), Centro Hospitalar e Universitário de Coimbra, Centro de Desenvolvimento da Criança, Coimbra, Portugal; ^9^Centro de Referência de Doenças Hereditárias do Metabolismo (DHM), Pediatria, Centro Hospitalar de São João, Porto, Portugal; ^10^Centro de Referência de Doenças Hereditárias do Metabolismo (DHM), Pediatria, Centro Hospitalar do Porto, Porto, Portugal; ^11^Centro de Referência de Doenças Hereditárias do Metabolismo (DHM), Medicina, Centro Hospitalar Lisboa Norte (CHLN), Lisbon, Portugal; ^12^Centre de Recerca e Investigació, Fundacio Hospital Sant Joan de Déu, Barcelona, Spain; ^13^Department of Pediatrics, University Medical Center, Johannes Gutenberg University, Mainz, Germany; ^14^Department of Pediatrics, University of Naples Federico II, Naples, Italy; ^15^Department of Chemistry and Biochemistry, Brigham Young University, Provo, UT, United States; ^16^Departamento de Ciências Médicas, Universidade de Aveiro, Aveiro, Portugal

**Keywords:** lipid antigen presentation, CD1b, CD1d, dendritic cells, monocytes, lysosomal storage diseases, natural killer T cells

## Abstract

The lysosome has a key role in the presentation of lipid antigens by CD1 molecules. While defects in lipid antigen presentation and in invariant Natural Killer T (iNKT) cell response were detected in several mouse models of lysosomal storage diseases (LSD), the impact of lysosomal engorgement in human lipid antigen presentation is poorly characterized. Here, we analyzed the capacity of monocyte-derived dendritic cells (Mo-DCs) from Fabry, Gaucher, Niemann Pick type C and Mucopolysaccharidosis type VI disease patients to present exogenous antigens to lipid-specific T cells. The CD1b- and CD1d-restricted presentation of lipid antigens by Mo-DCs revealed an ability of LSD patients to induce CD1-restricted T cell responses within the control range. Similarly, freshly isolated monocytes from Fabry and Gaucher disease patients had a normal ability to present α-Galactosylceramide (α-GalCer) antigen by CD1d. Gaucher disease patients' monocytes had an increased capacity to present α-Gal-(1-2)-αGalCer, an antigen that needs internalization and processing to become antigenic. In summary, our results show that Fabry, Gaucher, Niemann Pick type C, and Mucopolysaccharidosis type VI disease patients do not present a decreased capacity to present CD1d-restricted lipid antigens. These observations are in contrast to what was observed in mouse models of LSD. The percentage of total iNKT cells in the peripheral blood of these patients is also similar to control individuals. In addition, we show that the presentation of exogenous lipids that directly bind CD1b, the human CD1 isoform with an intracellular trafficking to the lysosome, is normal in these patients.

## Introduction

T lymphocytes can recognize lipid antigens presented by almost nonpolymorphic CD1 antigen-presenting molecules. In humans, CD1a, CD1b, CD1c, and CD1d are capable of presenting lipid antigens to T cells, while in mice only CD1d isoform is expressed. After synthesis, CD1 molecules traffic to the cell surface and are then internalized through the endocytic pathway. Along this recycling route, each CD1 isoform preferentially localizes in a different intracellular compartment, allowing them to encounter distinct lipid antigens (1). CD1a molecules mainly localize in early endosomes and possess the smallest groove, binding small lipids. CD1b traffics to lysosomes and is the isoform capable of binding larger lipids. CD1c and human CD1d have intermediate binding grooves and follow similar trafficking pathways, being localized in late endosomes. The trafficking pathway of mouse CD1d is similar to that used by human CD1b (2).

CD1-restricted T cells play important functions in the context of infection, immune response against tumors and autoimmunity (3). These cells can be classified by their recognition of antigens presented by group 1 CD1 molecules (CD1a, CD1b, and CD1c) or by CD1d. Broadly, group 1 CD1-restricted T cells are polyclonal and expand after antigen recognition in the periphery. CD1a-restricted T cells are among the most common self-reactive T cells in peripheral blood (4, 5), being abundant in skin where they are activated by Langerhans cells (4). CD1b-restricted T cells might recognize either microbial or self-lipids (6–10) although circulating self-reactive CD1b-restricted T cells have lower frequency (4, 5). Less consensual is the frequency of CD1c-restricted T cells (4, 5), which can be explained by a direct contact of the TCR with CD1c instead of the loaded lipids (11). Finally, CD1d-restricted T cells or Natural Killer T (NKT) cells, as they are also known because of their expression of NK and T cells surface markers, are divided into two subsets. Type I NKT or invariant NKT (iNKT) cells are characterized by the expression of a semi-invariant TCR (Vα24Jα18Vβ11 in humans and Vα14Jα18 paired with a limited repertoire of Vβ chains in mice) that recognizes the prototypic antigen α-galactosylceramide (α-GalCer). On the other hand, type II NKT cells express variable TCRs. While iNKT cells respond rapidly to both innate signals and TCR engagement producing large amounts of cytokines, some type II NKT cells display adaptive-like immune functions (5, 12).

Besides being the preferential intracellular compartment for CD1b and mouse CD1d localization, the lysosome contains hydrolytic enzymes and lipid-transfer proteins that process lipid antigens and assist the loading of lipids onto CD1 molecules, respectively (13–20). In addition, the low pH in this compartment induces relaxation of the CD1d structure, facilitating the loading of lipids (21). Indeed, the importance of the lysosome in lipid antigen presentation by mouse CD1d is reinforced by the defects described in mouse models of lysosomal storage diseases (LSDs) (22). LSDs are a group of individually rare inherited metabolic diseases characterized by the accumulation of specific macromolecules in the lysosome, including lipids, usually as a result of a deficiency in a lysosomal enzyme. There is no effective treatment for most LSDs. However, for some specific LSDs, enzyme replacement and/or substrate reduction therapies have been developed (23, 24). Lipid antigen presentation by CD1d is impaired in mouse models of several LSD including Sandhoff (25, 26), Niemann-Pick C (NPC) (16, 26–28), GM1 gangliosidosis (26, 28), and Fabry disease (26, 29). The alteration in lipid antigen presentation is accompanied by a defect in the percentage of both thymic and peripheral iNKT cells (16, 26–30). However, in NPC disease (31), Fabry disease (32), and Gaucher disease (33) patients' blood, no differences were observed in the frequency of total iNKT cells. Nevertheless, Fabry patients showed a reduction in the CD4^+^ and an increase in the double negative (DN) iNKT populations (32).

The abnormal accumulation of material in the lysosome/late endosomes of LSD patients is a key feature in LSD. However, there are several other cellular alterations described in the context of these diseases. In fact, impairment in endolysosomal trafficking (34, 35) has been described in several LSDs, namely NPC disease. In this regard, such impairment results in a greater accumulation of lipids in the late endosome (36). Moreover, defects in autophagy and lipid trafficking have also been reported (34, 37, 38) and, in Gaucher disease, an increase in lysosomal pH has been described (39), most likely impairing the relaxation of CD1 molecules' structure, thus hindering the loading of lipids in these molecules. Altogether, these cellular alterations may affect lipid antigen presentation by human CD1 molecules, particularly CD1b, which recycles through the lysosome (40).

However, studies of lipid antigen presentation in LSD patients are scarce; to date, the analysis of lipid antigen presentation in LSD patients is restricted to NPC disease where only CD1d-mediated presentation was studied using EBV transformed B cell lines as antigen presenting cells (APCs) (31). Herein, lipid antigen presentation by human CD1b and CD1d was studied in the context of four of the most common LSDs: Fabry, Gaucher, NPC, and MPS-VI diseases. These diseases represent LSD with distinct symptomatology and are characterized by the accumulation of different types of molecules in the lysosomes (Table 1). Gaucher and Fabry diseases are sphingolipidoses caused, respectively, by pathogenic mutations in GBA [encoding β-Glucosidase (GCase)] and GLA [encoding α-galactosidase A (α-Gal A)] genes (Table 1) and present as multisystemic disorders. Gaucher disease involves the visceral organs, bone marrow, and bone in almost all affected patients, whereas in Fabry disease involvement of the heart, kidney, and brain are the main sources of morbidity and premature death (41, 42). NPC disease is caused by defective transport of cholesterol (due to NPC1 and NPC2 genes' defects) with sequestration of unesterified cholesterol in lysosomes and late endosomes, and is clinically heterogeneous, mainly affecting the visceral organs (liver, spleen, and lungs) and the central nervous system (43). MPS VI is caused by arylsufatase B (ASB) enzyme deficiency and leads to the accumulation of dermatan sulfate and chondroitin sulfate (Table 1), which manifests as a multisystemic disorder affecting mainly the skeleton (44). The contribution of Type II NKT cells in the development of chronic B-cell activation and gammopathy in Gaucher disease has been proposed (33). For the other LSD, the contribution of CD1-restricted T cells to the clinical manifestation is not well defined. However, we could envision that they may be involved in LSD-associated chronic inflammation (34).

**Table 1:** Characteristics of lysosomal storage diseases studied.

**Disease**	**Protein defect**	**Material stored**
Fabry	α-Galactosidase A (α-Gal A)	Globotriaosylceramide (Gb3)
Gaucher	β-Glucosidase (GCase)	Glucosylceramide (GlcCer), Glucosylsphingosine (GlcSph)
Niemann-Pick disease (NPC)	Niemann-Pick C1 or C2 (NPC1/NPC2)	Unesterified cholesterol, Sphingolipids
Mucopolysaccharisodis type VI (MPS-VI)	Arylsulfatase B (ASB)	Dermatan sulfate, Chondroitin sulfate

In contrast to the data obtained in mice, no defects in lipid antigen presentation by CD1b or CD1d were observed in patients of this study, results that are also supported by the determination of normal frequencies of peripheral blood iNKT cells.

## Materials and Methods

### Biological Samples

Antigen presentation assays included 8 Fabry (only males), 16 Gaucher, 8 NPC (all with mutations in the NPC1 gene), 7 MPS-VI disease patients, and 52 healthy blood donors (all adults). Four Fabry and two Gaucher patients were not undergoing enzyme replacement therapy. The primary etiology and nature of the stored material for the four LSDs studied are depicted in Table 1. Table 2 indicates patient details and the correspondent codes used for their identification in the antigen presentation assays.

**Table 2:** Patients analyzed in antigen presentation assays.

**Disease**	**Code**	Age range^*^	**ERT/SRT**
Fabry	1F	51–55	Yes
	2F	36–40	Yes
	3F	61–65	Yes
	4F	51–55	Yes
	5F	46–50	No
	6F	36–40	No
	7F	16–20	No
	8F	21–25	No
Gaucher	1G	1–5	Yes
	2G	11–15	Yes
	3G	26–30	Yes
	4G	11–15	Yes
	5G	51–55	No
	6G	46–50	Yes
	7G	76–80	Yes
	8G	66–70	Yes
	9G	26–30	No
	10G	36–40	Yes
	11G	71–75	Yes
	12G	71–75	Yes
	13G	60–65	Yes
	14G	6–10	Yes
	15G	21–25	Yes
	16G	21–25	Yes
NPC	1N	1–5	Yes
	2N	16–20	Yes
	3N	16–20	Yes
	4N	6–10	Yes
	5N	21–25	Yes
	6N	26–30	Yes
	7N	31–35	Yes
	8N	11–15	Yes
MPS-VI	1M	16–20	Yes
	2M	16–20	Yes
	3M	26–30	Yes
	4M	11–15	Yes
	5M	16–20	Yes
	6M	16–20	Yes
	7M	5–10	Yes

* *Patient age was within this interval at the moment of the study*.

Studies of iNKT cell frequencies included 15 Fabry (8 males, 7 females, all adults), 19 Gaucher (8 males−1 pediatric, 11 females−3 pediatrics), 9 NPC (all with mutations in the NPC1 gene, 6 males−2 pediatrics, 3 females−1 pediatric), 13 MPS-VI disease patients (6 males−2 pediatrics, 7 females−3 pediatrics), and 92 healthy blood donors (77 adults−37 males and 40 females, 15 children−12 males and 3 females). Patients and controls ≥16 years old were considered as adults.

Written informed consent was obtained from all patients enrolled in the study in accordance with the Helsinki declaration. The study was approved by local ethical committees (from the hospitals where patients and controls were recruited) and by the national commission of data protection. Patients were recruited from São João Hospital, Porto—Portugal; Santo António Hospital, Porto—Portugal; Santa Maria Hospital, Lisbon—Portugal; Senhora da Oliveira Hospital, Guimarães—Portugal; Pediatric Coimbra Hospital, Coimbra—Portugal; Sant Joan de Déu Hospital, Barcelona—Spain; Federico II University Hospital, Naples—Italy; and University Children's Hospital, Mainz—Germany. All but one of the NPC patients were under substrate reduction therapy (SRT). Most of the Fabry, Gaucher and MPS-VI disease patients analyzed were under enzyme replacement therapy (ERT). In Table 2; **Figure 6**, and Supplementary Figure 1 it is indicated whether the patients are under treatment or not. Fabry and Gaucher patients received ERT by infusion every 2 weeks, while MPS VI patients were treated intravenously with ERT weekly. NPC patients received oral daily substrate reduction therapy. Blood samples from Fabry, Gaucher, and MPS-VI disease patients were always collected before treatment infusion to minimize the putative effect of treatments in the analyzed cells. The adult control subject population was composed of healthy blood donors from the Instituto Português do Sangue, Porto—Portugal, or from the Immuno-hemotherapy department of São João Hospital. Control pediatric subjects were recruited among children undergoing orthopedic surgery at São João Hospital, without infections, underlying chronic illness, or taking medication.

### Peripheral Blood Mononuclear Cells Isolation (PBMCs), Monocytes Purification, and Mo-DCs Generation

PBMCs were separated by Histopaque-1077® (Sigma-Aldrich, St. Louis, MO, USA) density centrifugation following the manufacturer's instructions. Monocytes were isolated by positive selection with anti-CD14 magnetic beads using the MACS cell separation system (Miltenyi Biotec, Cologne, Germany).

CD14^+^ cells were used after purification (monocytes) or to promote differentiation in dendritic cells by plating them at 10^6^ cells/mL in RPMI 10% iFBS supplemented with 50 ng/mL of IL-4 and GM-CSF (ImmunoTools, Friesoythe, Germany). After 7 days, monocyte-derived dendritic cells (Mo-DCs) were collected and used for lipid antigen presentation assays.

### Flow Cytometry

Monocyte purity, proper Mo-DC differentiation and the basal state of activation of both cells were assessed by flow cytometry using the following anti-human monoclonal antibodies: CD14 (M5E2, Biolegend, San Diego, CA, USA), CD1b (SN13, Biolegend), CD1d (51.1, Biolegend), CD11c (3.9, eBioscience), CD80 (2D10, Biolegend), HLA-DR (LN3, eBioscience).

iNKT and T cell determinations were performed in total PBMCs or in CD14^−^ fractions, using CD1d-PBS57 tetramers (NIH Tetramer Core Facility, Emory University, Atlanta, GA, USA) and the following anti-human monoclonal antibodies: CD3 (OKT3, eBioscience), CD4 (OKT4, Biolegend), and CD8 (RPA-T8, eBioscience). The purity of T cell clones VM-D5 and JS63 was assessed by using CD1d-PBS57 tetramers (NIH Tetramer Core Facility, Emory University, Atlanta, GA, USA) together with anti-human CD3 (OKT3, eBioscience) monoclonal antibody. The purity of T cell clones s33d, GG33A, and DS1C9b was assessed by using anti-human TCR Vβ13.1 (IMMV222), Vβ18 (BA62.6), and Vβ7.1 (ZOE), monoclonal antibodies from Immunotec (Immunotec Research Inc, Canada). Cells were acquired in a FACS Canto II (BD Biosciences, San Diego, CA, USA) using the BD FACSDiva™ software (BD Biosciences). Data analysis was performed with FlowJo® v10 (FlowJo LLC, Ashland, OR, USA).

### Generation of Fabry and Gaucher Disease *in vitro* Models

Generation of the Fabry and Gaucher disease *in vitro* cell models was adapted from previously described protocols (45, 46). To induce Globotriaosylceramide (Gb3) accumulation, CD1-transfected C1R cells were cultured for 72 h in the presence of 1 mM deoxynojirimycin (DGJ, Sigma-Aldrich) alone or together with 10 μM Gb3 (Matreya, LLC, Pleasant Gap, PA, USA) in complex with fatty acid-free bovine serum albumin (BSA) (Sigma-Aldrich). Glucosylceramide (GlcCer) accumulation in CD1-transfected C1R cells was achieved by a 72 h culture with 1 mM conduritol B epoxide (CBE, Sigma-Aldrich). Treated and untreated cells were collected and used for lipid antigen presentation assays or pelletized. Pellets were analyzed by Thin-layer chromatography (TLC).

### Thin-Layer Chromatography

Thin-layer chromatography of neutral lipids from CD1-transfected C1R cell pellets was performed as described (47). To overcome plate loading differences, the intensity of GlcCer and Gb3 bands was divided by the intensity of the Sph/Gb4 band. The lipid standards used were the following: glucosylceramide (GlcCer); phosphatildylethanolamine (PE); lactosylceramide (LacCer) and sphingomyelin (Sph) from Sigma-Aldrich; globotriaosylceramide (Gb3) from Nacalai Tesque Inc.; ganglioside GM1 (Cer4) from Calbiochem; and globotetrahexosylceramide (Gb4) from Matreya.

### T Cell Clone/iNKT Cell Line Culture and Re-stimulation

The maintenance of T cell clones and of the iNKT cell line was performed as previously described (48). The following T cell clones were used in the assays: DS1C9b (CD1b-restricted, sulfatide-specific) (49); GG33A (CD1b-restricted, GM1-specific) (48); JS63 (CD1d-restricted, α-GalCer-specific) (50); VM-D5 (CD1d-restricted, α-GalCer-specific) (50) and s33d (CD1d-restricted, sulfatide-specific) (50). An iNKT cell line (51) was also used.

### Lipid Antigen Presentation Assays

Monocytes, Mo-DCs or CD1-transfected C1R cells were cultured with sulfatide (30–0.04 μg/mL, Sigma-Aldrich), GM1 (50–0.07 μg/mL, Sigma-Aldrich), α-Galactosylceramide (α-GalCer) (at 50 ng/mL or 50–3.12 ng/mL, Avanti polar lipids, Alabaster, AL, USA) or α-Gal-(1-2)-αGalCer (300–50 ng/mL). Lipids, with the exception of α-GalCer, were first dissolved in methanol or PBS 0.5% Tween 20 and then diluted in non-supplemented RPMI to have a maximum of 1% vehicle in culture. α-GalCer was resuspended in PBS and directly diluted in non-supplemented RPMI. After 4 h, an iNKT cell line or T cell clones were added and 40 h later, supernatants were collected for cytokine production determination by ELISA. The following antibody pairs from Biolegend were used: purified anti-human GM-CSF (BVD2-23B6) and biotinylated anti-human GM-CSF (BVD2-21C11); purified anti-human IL-4 (8D4-8) and biotinylated anti-human IL-4 (MP4-25D2).

### Statistics

Data distribution of the frequencies of iNKT cells (total, CD4^+^, CD8^+^, DN) and T cells (total, CD4^+^, CD8^+^) from adult and child healthy controls was assessed using the D'Agostino & Pearson test. Then, an unpaired *t*-test (normal distribution) or a Mann-Whitney test (non-normal distribution) was used to compare adult and child healthy control populations. As no significant differences were observed (results not shown), data from adults and children were pooled.

To compare the frequencies of iNKT cells (total, CD4^+^, CD8^+^, DN) and T cells (total, CD4^+^, CD8^+^) from LSD patients and healthy controls, data normality was first checked using the D'Agostino & Pearson test. When all the populations studied passed the normality test, an ordinary one-way ANOVA was used to compare each group of patients with controls. When the populations analyzed did not pass the normality test, the Kruskal-Wallis test was used. *P*-values lower than 0.05 were considered significant.

Statistical analysis of lipid antigen presentation assays was performed by comparing the amount of cytokine produced in the coculture of APC with T cells between LSD patients and control subjects. A single concentration of antigen was used for this purpose. Data normality was first checked using the Shapiro–Wilk test. Then, an unpaired *t*-test (normal distribution) or a Mann-Whitney test (non-normal distribution) was used to compare the two groups. *P*-values lower than 0.05 were considered significant. Along with the comparison of the raw data, and due to the inter-experimental variation, comparison was also done after normalization of the values of cytokine production for each independent experiment. The values of cytokine production were relativized considering 100 as the highest cytokine production value within each experiment for the chosen antigen concentration. All the analyses were performed using GraphPad Prism software v7.04 (GraphPad Software Inc., CA, USA).

## Results

### Mo-DCs From Fabry, Gaucher, NPC and MPS-VI Disease Patients Do Present Antigens by CD1b

*in vitro* differentiation of monocytes to dendritic cells (Mo-DCs) is accompanied by an increase in the expression of CD1b (52). To study antigen presentation by CD1b, the capacity of Mo-DCs from Fabry, Gaucher, NPC (with mutations in NPC1) and MPS-VI disease patients to present lipids by CD1b was analyzed and compared with that of healthy controls. The expression of CD80 and CD1b on the surface of Mo-DCs from patients and controls was assessed after the differentiation process and prior to the experiments. Mo-DCs from LSD patients had similar levels of CD1b and CD80 cell surface expression to those of control subjects (Supplementary Table 1).

Two previously described CD1b restricted T cell clones were used: the GG33A T cell clone, specific for GM1 (48), and the DS1C9b T cell clone, sulfatide specific (49). The GG33A T cell clone carries the Vβ18 TCR chain while the DS1C9b T cell clone expresses the Vβ7.1 TCR chain (personal communication from Lucia Mori and Gennaro de Libero who produced the T cell clones). The identity of the T cell clones was confirmed by flow cytometry (Supplementary Figure 2).

**Figure 1:**
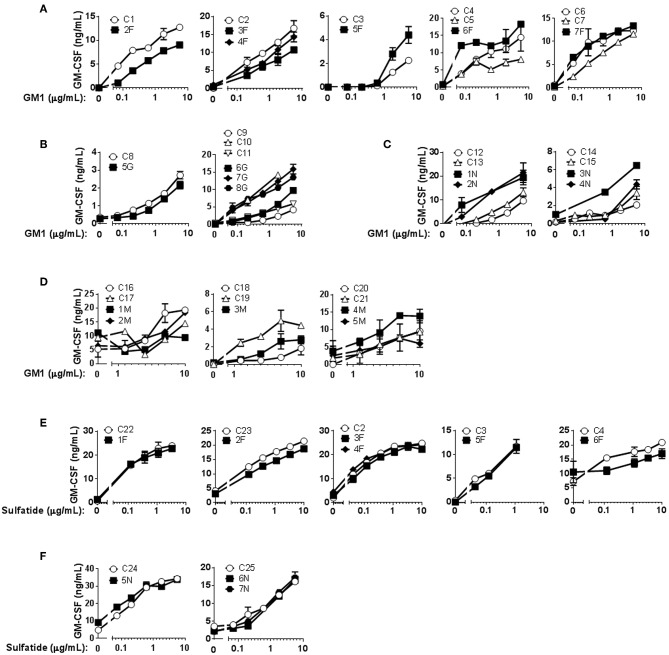
CD1b-restricted lipid antigen presentation by Mo-DCs from Fabry, Gaucher, NPC and MPS-VI disease patients. Mo-DCs from Fabry **(A)**, Gaucher **(B)**, NPC **(C)**, and MPS-VI **(D)** disease patients and control subjects were loaded with graded doses of GM1 and co-cultured with the CD1b-restricted T cell clone GG33A. Mo-DCs from Fabry **(E)** and NPC **(F)** disease patients and control subjects were loaded with graded doses of sulfatide and co-cultured with the CD1b-restricted T cell clone DS1C9b. T cell response was analyzed by measuring GM-CSF release to the supernatant by ELISA. Patients are represented with filled symbols and control subjects with open symbols. Each symbol represents mean ± SD of duplicates for the same individual at the indicated antigen concentration. Each graph corresponds to an independent experiment.

Mo-DCs from Fabry (Figure 1A), Gaucher (Figure 1B), NPC (Figure 1C), and MPS-VI (Figure 1D) disease patients were capable of presenting the exogenously added antigen GM1 to the CD1b-restricted T cell clone GG33A (Supplementary Figure 3). Curiously, Mo-DCs from NPC disease patients seem to have a higher presentation capacity of the antigen GM1 when comparing with control subjects. This difference was statistically significant (*P* = 0.0041) for the normalized values of cytokine production in the presence of 5 μg/mL of GM1 (Supplementary Figure 3A). Regardless of the higher presentation capacity observed in NPC patients to present GM1 through CD1b, the same was not observed using the sulfatide-specific CD1b-restricted DS1C9b T cell clone (Figure 1F and Supplementary Figure 3B). Sulfatide presentation through CD1b was also analyzed in Fabry patients' Mo-DCs, and a small decrease in the capacity to activate the DS1C9b clone was observed (Figure 1E) when considering one of the tested concentrations (1 μg/mL of sulfatide, *P* = 0.0456) (Supplementary Figure 3B).

**Figure 2:**
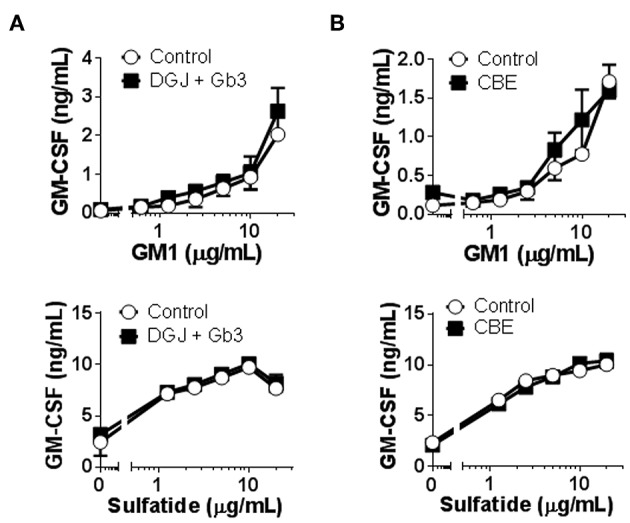
CD1b-restricted lipid antigen presentation by *in vitro* cell models of Fabry and Gaucher diseases. **(A)** Fabry disease *in vitro* cell model: CD1b-transfected C1R cells were treated with DGJ 1 mM + Gb3:BSA (filled symbols) or remained untreated (empty symbols). **(B)** Gaucher disease *in vitro* cell model: CD1b-transfected C1R cells were treated with CBE (filled symbols) or remained untreated (empty symbols). Then, C1R cells were loaded with graded doses of GM1 or sulfatide and co-cultured with the CD1b-restricted T cell clone GG33A or DS1C9b, respectively. T cell response was analyzed by measuring GM-CSF release to the supernatant by ELISA. Each symbol represents mean ± SD of duplicates for the same individual at the indicated antigen concentration. One representative experiment out of at least two is shown.

To overcome the high degree of variability observed when testing patients as well as control subjects, and to assure that lipid storage was indeed occurring, we used cellular models of Fabry (45) and Gaucher (46) diseases. C1R cells expressing CD1b were treated with DGJ, an inhibitor of α-galactosidase A (α-Gal A) (the enzyme deficient in Fabry disease) alone or together with Gb3 (the main storage material of Fabry disease) complexed with BSA, or with CBE, an inhibitor of the enzyme β-glucosidase (GCase) (deficient in Gaucher disease). TLC analyses confirmed that simultaneous treatment with DGJ and Gb3:BSA induced Gb3 accumulation (Supplementary Figure 4A) and that culture with CBE induced glucosylceramide (GlcCer) storage (Supplementary Figure 4B). These cells were then used as APCs in antigen presentation assays. No differences were observed when cells accumulating Gb3 (Figure 2A) or GlcCer (Figure 2B) were compared with untreated cells for their capacity to present the antigens by CD1b.

In conclusion, no major differences were found in CD1b-restricted presentation of GM1 or sulfatide antigens to specific T cell clones between healthy subjects and LSD patients.

### Mo-DCs From Fabry, Gaucher, NPC and MPS-VI Disease Patients and Fabry and Gaucher Fresh Monocytes, Do Present Antigens by CD1d

Several LSD animal models demonstrated defects in CD1d-mediated lipid antigen presentation to iNKT cells, including Fabry and NPC disease mouse models (16, 26–29). Type II NKT cells also constitute an important part of CD1d-restricted T cells in humans (53). We analyzed the capacity of Mo-DCs from Fabry, Gaucher, NPC, and MPS-VI disease patients to activate the iNKT cell clone JS63 in response to α-GalCer and the type II NKT cell clone s33d (50) in response to sulfatide by measuring cytokine production by ELISA. The s33d T cell clone carries the TCR Vβ13.1 (50), which was used to confirm the identity of the s33d T cells by flow cytometry (Supplementary Figure 2). Analysis of CD1d and CD80 expression by Mo-DCs revealed no significant differences between control subjects and LSD patients (Supplementary Table 1). In Supplementary Figure 5 we analyzed cytokine production in response to antigen stimulation in Mo-DCs-iNKT cell cocultures. Cytokine production was also measured in parallel cultures of Mo-DCs only and Mo-DCs plus lipid antigen without iNKT cells. As expected, iNKT cells responded to α-GalCer loaded Mo-DCs with high production of GM-CSF. However, Mo-DCs cultured alone were also able to produce some GM-CSF, with or without lipid antigen stimulation.

**Figure 3:**
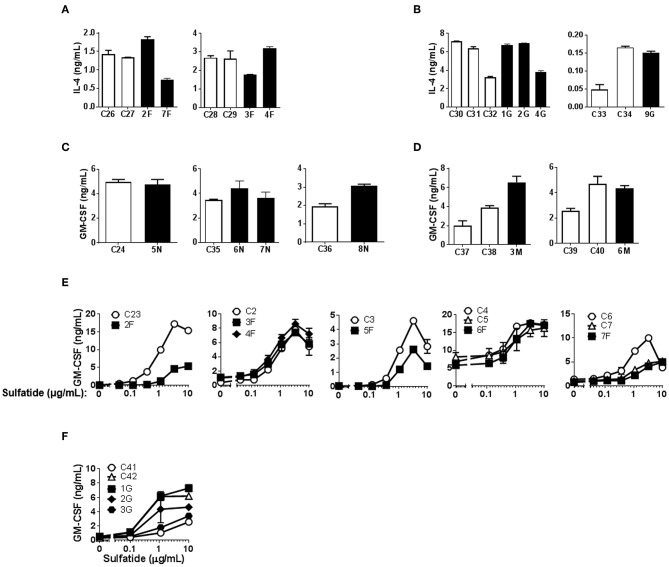
CD1d-restricted lipid antigen presentation by Mo-DCs from Fabry, Gaucher, NPC and MPS-VI disease patients. Mo-DCs from Fabry **(A)**, Gaucher **(B)**, NPC **(C)**, and MPS-VI **(D)** disease patients and control subjects were loaded with 50 ng/mL of α-GalCer and co-cultured with the iNKT cell clone JS63. T cell response was analyzed by measuring IL-4 or GM-CSF release to the supernatant by ELISA. Patients are represented with filled columns and control subjects with open columns. Each column represents mean ± SD of duplicates for the same individual. Mo-DCs from Fabry **(E)** and Gaucher **(F)** disease patients and control subjects were loaded with graded doses of sulfatide and co-cultured with the type II NKT cell clone s33d. T cell response was analyzed by measuring GM-CSF release to the supernatant by ELISA. Patients are represented with filled symbols and control subjects with open symbols. Each symbol represents mean ± SD of duplicates for the same individual at the indicated antigen concentration.

α-GalCer presentation by Fabry, Gaucher, NPC, and MPS-VI Mo-DCs was successful, although some variation in the degree of activation could be observed among both control subjects and LSD patients (Figures 3A–D). The presentation of sulfatide to type II NKT cells by Mo-DCs from Fabry and Gaucher patients was also studied (Figures 3E,F). No statistically significant differences were observed in the capacity of Mo-DCs from patients and controls to present α-GalCer and sulfatide to iNKT cells and type II NKT cells, respectively (Supplementary Figure 6).

**Figure 4:**
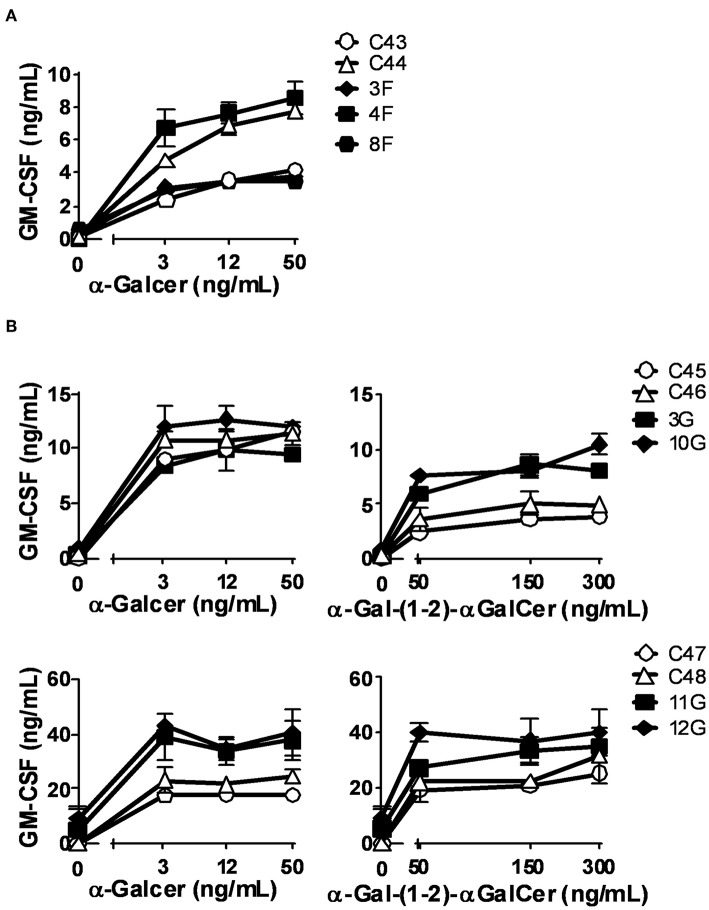
CD1d-restricted lipid antigen presentation by monocytes from Fabry and Gaucher disease patients. Monocytes from Fabry **(A)** and Gaucher **(B)** disease patients and control subjects were loaded with graded doses of α-GalCer (Fabry and Gaucher) or α-Gal-(1-2)-α-GalCer (Gaucher) and co-cultured with an iNKT cell line. Patients are represented with filled symbols and control subjects with open symbols. Each symbol represents mean ± SD of duplicates for the same individual at the indicated antigen concentration.

Modifications of the cellular lipid content and in CD1d expression occur during the process of *in vitro* DC differentiation from monocytes (52). Therefore, Mo-DCs might not be representative of the lipid antigen presentation occurring *in vivo* in patients. To overcome this issue, we used fresh monocytes from Fabry and Gaucher patients in addition to control subjects as APC in activation assays with an iNKT cell line (51) using α-GalCer (Fabry and Gaucher) and α-Gal-(1-2)-αGalCer (Gaucher) as antigens. α-Gal-(1-2)-αGalCer only becomes antigenic after cleavage in the lysosome by α-Gal A, the enzyme deficient in Fabry disease patients, meaning that the loading into CD1d preferentially happens inside the cell. Monocytes from LSD patients had similar levels of CD1d and CD80 cell surface expression to those of control subjects (Supplementary Table 2). Similar to what was observed when Mo-DCs were used, a variation in the T cell response was observed between subjects (Figure 4). Nevertheless, monocytes from Fabry (Figure 4A) and Gaucher patients (Figure 4B) were able to activate the iNKT cell line within the range of control monocytes when stimulated with α-GalCer. Monocytes from Gaucher disease patients (Figure 4B) have a higher capacity of α-Gal-(1-2)-αGalCer presentation when compared with control subjects. This difference was statistically significant (*P* = 0.005) for the normalized values of cytokine production in the presence of 50 ng/mL of the antigen (Supplementary Figure 7).

**Figure 5:**
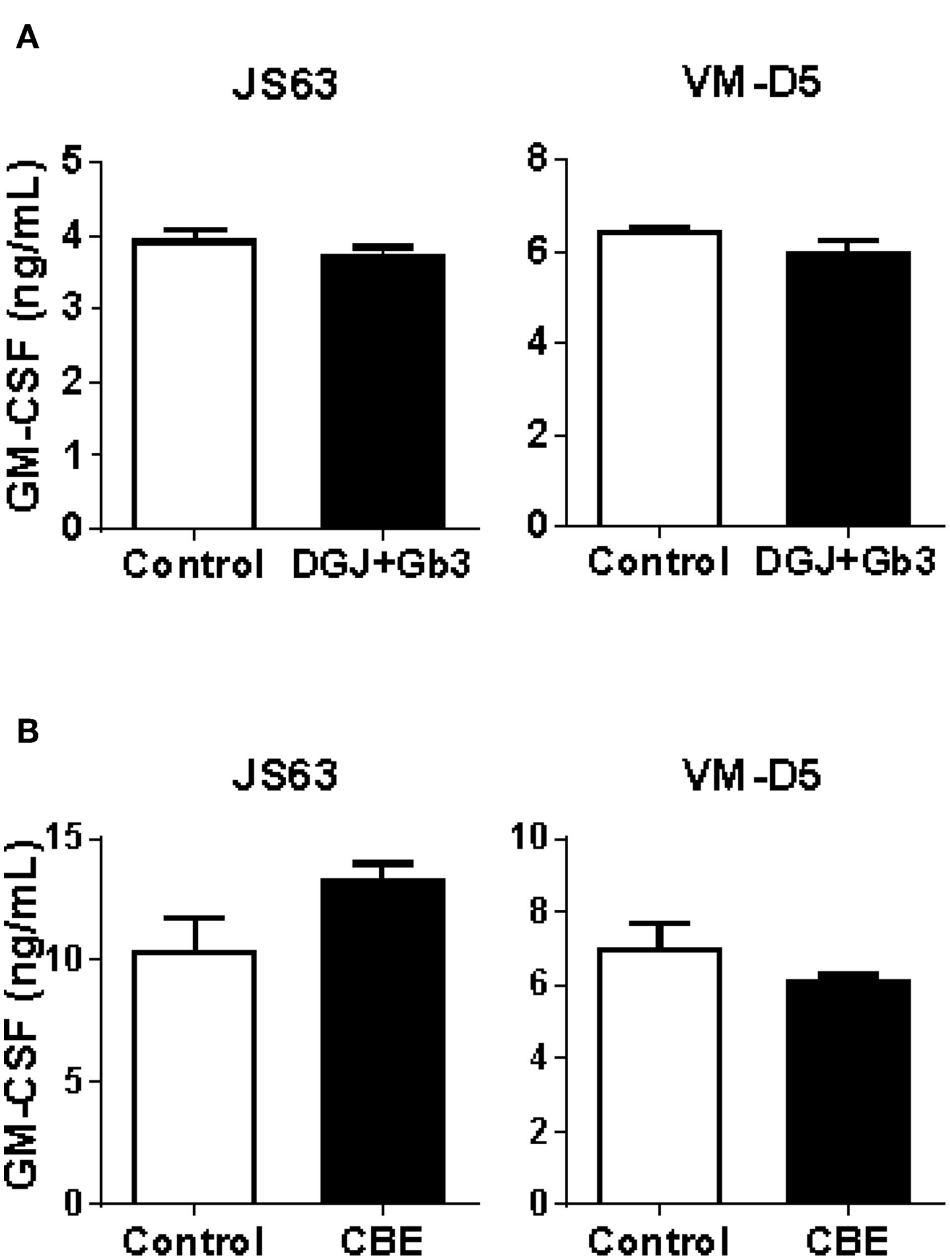
CD1d lipid antigen presentation by *in vitro* cell models of Fabry and Gaucher diseases. **(A)** Fabry disease *in vitro* cell model: CD1d-transfected C1R cells were treated with DGJ 1 mM + Gb3:BSA (filled columns) or remained untreated (empty columns). **(B)** Gaucher disease *in vitro* cell model: CD1d-transfected C1R cells were treated with CBE (filled columns) or remained untreated (empty columns). Then, C1R cells were loaded with 50 ng/mL of α-GalCer and co-cultured with the iNKT cells clones JS63 or VM-D5, or with an iNKT cell line. T cell response was analyzed by measuring GM-CSF release to the supernatant by ELISA. Each column represents mean ± SD of duplicates. One representative experiment out of at least two is shown.

We confirm the results of α-GalCer presentation, in a system that eliminates individual donor variability, and we tested the capacity of Gb3- and GlcCer-loaded C1R cells expressing CD1d to present α-GalCer by CD1d. We found that both Fabry (Figure 5A) and Gaucher (Figure 5B) *in vitro* APC models had similar abilities to activate iNKT cells, in comparison with unloaded cells. Thus, APCs from Fabry, Gaucher, NPC, and MPS-VI disease patients are capable of presenting lipid antigens that bind CD1d.

### Fabry, Gaucher, NPC and MPS-VI Disease Patients Present Normal Frequencies of Circulating iNKT Cells

**Figure 6:**
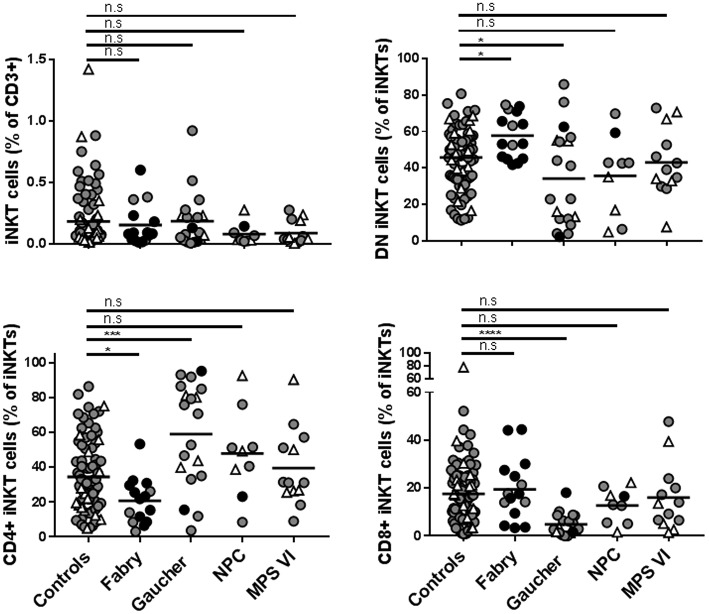
Frequencies of total iNKT cells and DN, CD4^+^, and CD8^+^ iNKT cells in Fabry, Gaucher, NPC, and MPS-VI disease patients. iNKT cells were identified in PBMCs or CD14^−^ fractions obtained from Fabry, Gaucher, NPC, and MPS-VI disease patients or control subjects, by their expression of CD3 and the recognition of the CD1d-PBS57 tetramer. Antibodies against CD4 and CD8 were also used to define DN and positive subsets. Circles represent adults (≥16 years-old) and triangles represent children (those under 16 years of age). Black circles identified adult patients that were not under treatment. All the pediatric patients were receiving treatment. Horizontal line represents the mean of each group studied. Data normality was analyzed using the D'Agostino & Pearson normality test. To compare patients with the control population, one-way ANOVA (for data with normal distribution) or Kruskal-Wallis test (data with non-normal distribution) were used. ^*^p ≤ 0.05, ^***^p ≤ 0.001, ^****^p ≤ 0.0001.

The frequency of iNKT cells is reduced in different mouse models of LSD (16, 22, 25–30). The same was not observed in NPC (31), Fabry (32), and Gaucher disease (33) patients' blood. Here, we studied the percentage of total iNKT cells and their CD4/CD8 phenotype in the blood of Gaucher, NPC, and MPS-VI disease patients compared with control individuals and our own published data on Fabry disease patients (32). No significant differences were observed in the percentage of total iNKT cells in Gaucher, NPC, and MPS-VI disease patients' blood (Figure 6). However, when CD4/CD8 expression was analyzed, Gaucher disease patients showed an increase in the percentage of CD4^+^ and a decrease in the percentage of DN and CD8^+^ iNKT cells. NPC and MPS-VI disease patients did not present significant alterations in the frequencies of CD4/CD8/DN iNKT cells. Differences observed in Gaucher disease patients were iNKT cell-specific, as total T cells and their subsets remained unaltered (Supplementary Figure 1). Therefore, although Fabry and Gaucher disease APCs showed no defects in their antigen presentation capacity by CD1d, enzyme deficiency and their consequences could impact the presence of certain iNKT cell populations in the peripheral blood of these patients.

## Discussion

The lysosome has been described as an important intracellular compartment for lipid antigen presentation, emphasized by the defects observed in several mouse models of LSD (16, 25–29). However, the effects of lysosomal dysfunction present in LSD patients on lipid antigen presentation by different human CD1 molecules are unclear. Herein, we analyzed lipid antigen presentation in patients affected by four different LSDs, including three sphingolipidoses (Fabry, Gaucher, and NPC) and one mucopolysaccharidosis (MPS-VI). We focused on CD1b, as it is the human CD1 molecule that upon internalization recycles to the lysosome, and on CD1d, to compare with the published data on mouse models of LSD.

We found no major alterations in the capacity of APCs from Fabry, Gaucher, NPC, and MPS-VI disease patients to present exogenously added antigens that bind CD1b. The same was observed for CD1d presentation of α-GalCer in the four studied LSD. This is in contrast with what was observed for CD1d-mediated lipid antigen presentation in mouse models of several LSDs (16, 26–29). α-GalCer is a lipid whose internalization into APCs is mediated by scavenger receptors (54) and is presented by CD1d at the cell surface. Moreover, the loading of α-GalCer onto CD1d is influenced by its access to a functional lysosomal compartment in human APCs (55) and, in line with this notion, several studies have described that APCs from mouse models of LSD yield a decreased capacity to present α-GalCer (16, 26–29).

The capacity of Gaucher disease patients' monocytes to present α-Gal-(1-2)-αGalCer seems to be elevated, which is also in contrast with the observation of a decreased capacity of APCs from LSD mouse models to present α-Gal-(1-2)-αGalCer (16, 25–27), a lipid that needs to be internalized and cleaved by the lysosomal α-Gal A enzyme to become antigenic.

In the NPC1 mouse model, lipid antigen trafficking to the lysosome is compromised (27), which might explain the defects in lipid antigen presentation. It would be interesting to address if this impairment also occurs in APCs from LSD patients, especially since lipid traffic in LSD patients' fibroblasts was shown to be altered (34).

In accordance with our results, a previous report has shown that EBV transformed B cell lines from NPC patients, and transfected with CD1d, have a normal capacity to present lipids by this molecule (31). These apparent discrepancies between mouse and human CD1d lipid antigen presentation in NPC and Fabry diseases could be related to: (i) the described differences in intracellular trafficking between human and mouse CD1d; (ii) the discrepancy in lysosomal function between LSD patients and mouse models; (iii) the different type of APC used in mouse and human studies.

Mouse CD1d mainly recycles to the lysosome, contrary to human CD1d which is usually found in late endocytic compartments (56). In fact, when B cell lines from NPC patients were transfected with mouse CD1d instead of human CD1d, a decrease was observed in the APC capacity to present α-Gal-(1-2)-αGalCer (31).

CD1b mediated antigen presentation was analyzed using GM1 and sulfatide, which are lipids that can bind to the cell surface of CD1b (49, 57). GM1, due to its similarities to GM3, could also require internalization via scavenger receptors in order to be presented by CD1b (54). However, we must note that GM1 has also been found to bind CD1b at the surface of fixed cells (49).

Interestingly, we observed that despite the lysosomal alterations occurring in LSD patients, lipid presentation through CD1b was not affected, allowing us to conclude that surface CD1b in LSD APC is competent in presenting lipids. Therefore, to draw more robust conclusions about CD1b-mediated lipid antigen presentation, it would be useful to perform the same type of studies with antigens that can only be presented by CD1b upon internalization, like some *Mycobacterium* lipids (6).

Another possible explanation for the differences found between mice and humans is that while LSD animal models correspond to full knock-outs of the gene, deficiencies in humans may not translate into a full absence of the protein function. It is also possible that partial restoration of enzymes or their products with LSD treatments could be sufficient to compensate for the potential impact on antigen presentation. However, in the present study, blood (with the exception of NPC patients who received oral daily substrate reduction therapy) was always collected before infusion of the treatment (that was done weekly or every 2 weeks) and dendritic cell differentiation from monocytes takes more than 6 days in culture. It is therefore unlikely that the recombinant enzyme used in the infusions was present in considerable amounts in the Mo-DCs used in the antigen presentation assays. Moreover, no difference in the antigen presentation capacity was observed between treated and non-treated patients.

Lipid antigen presentation defects observed in LSD mouse models seem to be dependent on the nature of APC. While splenocytes and thymocytes from GM1 gangliosidosis, splenocytes from Sandhoff, or splenic DCs from Fabry disease mice showed defects in α-GalCer presentation, bone marrow derived DCs (BM-DCs) from the same mice were competent in presenting this antigen to iNKT cells (26, 28, 29, 58). These differences could be related to variable degrees of lipid storage in distinct APCs or biological differences in antigen uptake or presentation. It is known that fetal bovine serum used in culture has a deficit in essential fatty acids, which results in an increased lipogenesis by cells, leading to lipidomic modifications (59). This could justify the absence of alterations observed when using cells in culture, such as BM-DCs, Mo-DCs, or EBV-transformed B cell lines. Having this in mind, we tested the capacity of freshly isolated monocytes from Fabry and Gaucher patients to present antigens by CD1d. No major defects were observed in the presentation of the antigen α-GalCer, and monocytes from Gaucher disease patients present a slightly higher capacity to present α-Gal-(1-2)-αGalCer. Considering specifically Fabry disease patients, the lack of major lipid antigen presentation alterations can also be explained by the double effect of α-Gal A, the enzyme deficient in this disease. α-Gal A is known for its role in the degradation of Gb3, a lipid that we recently demonstrated to be inhibitory for iNKT cells (51). However, α-Gal A was also implicated in the degradation of α-psychosine, an antigen that was shown to be present in mammalian tissues (60). Thus, this enzyme is responsible for the degradation of both antigenic (α-psychosine) and inhibitory (Gb3) lipids for iNKT cells. Therefore, it is plausible that α-Gal A deficiency leads to an increase in the cellular content of both lipids, thus maintaining their ratio and preventing major alterations in iNKT cell activation.

In our study, we also found a variable response of T cell clones to Mo-DCs and monocytes from different subjects, which does not seem to be related to age or gender. CD1 genes display a limited polymorphism but some substitutions have been described for exon 2 (61), although the impact of these substitutions in lipid antigen presentation is not clear. Nevertheless, studies in mice have revealed that CD1d polymorphisms affect antigen presentation and activation of CD1d-restricted T cells (62, 63), suggesting that the same may happen in humans.

The percentage of total iNKT cells was found unaltered in the peripheral blood of Fabry (32), Gaucher, NPC, and MPS-VI disease patients. However, as described before for Fabry disease patients (32), an alteration in the frequency of CD4/CD8/DN iNKT cells was observed in Gaucher disease patients, showing a significant increase of the CD4^+^ population and a decrease of DN and CD8^+^ iNKT cells. These results suggest a higher egress of CD4^+^ iNKT cells from the thymus of Gaucher patients, which can be related with a propitious cytokine environment for the selection of this subset or an alteration in the antigen availability that could potentiate their selection. Finally, a difference in the frequency of iNKT cells in other localizations than blood cannot be excluded.

In conclusion, we show that APCs from Fabry, Gaucher, NPC, and MPS-VI disease patients are capable of presenting exogenous lipid antigens that bind CD1b and CD1d. In the future, for CD1b, it will be important to extend the study to lipid antigens that require internalization. It will be also interesting to elucidate whether lipid imbalances and other cellular alterations present in LSD will affect CD1 mediated self-lipid antigen presentation. Moreover, deciphering the factors favoring the thymic selection of CD4^+^ iNKT cells or inhibiting the proliferation of CD4^−^ iNKT cells in the periphery of Gaucher patients would also be relevant.

## Ethics Statement

Informed written consent was obtained from all patients in accordance with the Helsinki declaration. The study was approved by local ethical committees (from the hospitals were patients and controls were recruited) and by the national commission of data protection. Patients were recruited from São João Hospital, Porto—Portugal; Santo António Hospital, Porto—Portugal; Santa Maria Hospital, Lisbon—Portugal; Senhora da Oliveira Hospital, Guimarães—Portugal; Pediatric Coimbra Hospital, Coimbra—Portugal; Sant Joan de Déu Hospital, Barcelona—Spain; Federico II University Hospital, Naples—Italy; and University Children's Hospital, Mainz—Germany.

## Author Contributions

CP, BP-C, and HR performed the experiments, analyzed and interpreted the data and wrote the manuscript. MLM and AD performed the experiments and analyzed data. PA wrote the manuscript, recruited and clinically evaluated the patients. MC, PC-C, OA, MF, PG, ER, MP, YA, SF, EM, and EL-T recruited and clinically evaluated the patients. SD and PS synthesized the α-Gal-(1-2)-αGalCer lipid. MFM designed and coordinated the study, analyzed and interpreted data and wrote the paper. All authors revised and approved the manuscript.

### Conflict of Interest Statement

MFM has received a research grant from Sanofi-Genzyme, that partially funded this work. Sanofi-Genzyme had no role in study design and collection, analysis, and interpretation of the results. SF received consultancy fees/honoraria from Alexion Pharma GmbH and Orphan Europe and received travel expenses/accommodation for conference/meeting participation from Sanofi-Genzyme, Shire, Actelion, and PIAM. OA received research grants from Shire Human Genetic Therapies, Inc. and travel/accommodation grants for conference participation from Shire Human Genetic Therapies, Inc, Sanofi-Genzyme, Amicus, and Alexion Pharma GmbH. PG received travel expenses/accommodation for conference/meeting participation from Sanofi-Genzyme, Shire, Biomarin. PC-C received consultancy and travel expenses for conference participation from Shire. EL-T received speaker fees from Biomarin and travel expenses for conferences, from Sanofi and Biomarin. PA received research grants from Shire Pharmaceuticals and honoraria from Shire Pharmaceuticals, Sanofi-Genzyme, Amicus, Biomarin, and Ultragenyx. The remaining authors declare that the research was conducted in the absence of any commercial or financial relationships that could be construed as a potential conflict of interest.

## Supplementary Material

Supplementary Figure 1 Frequencies of total CD3^+^ T cells and their CD4^+^ and CD8^+^ subsets in Fabry, Gaucher, NPC and MPS-VI disease patients. Total T cells were identified, in PBMCs or CD14^−^ fractions obtained from Fabry, Gaucher, NPC and MPS-VI disease patients or control subjects, by their expression of CD3. Antibodies against CD4 and CD8 were also used to define positive subsets. Circles represent adults (over 16 years of age) and triangles children (under 16 years of age). Black circles identify adult patients that were not under treatment. All the pediatric patients were receiving treatment. Horizontal line represents the mean of each group studied. Normality of each group was analyzed using the D'Agostino & Pearson normality test. To compare patients with the control population, one-way ANOVA (for data with normal distribution) or Kruskal-Wallis test (data with non-normal distribution) were used. ^**^*p* ≤ 0.01.

Supplementary Figure 2 Determination of T cell purity. DS1C9b, GG33A, and s33d T cells purity was analyzed by using the anti-human TCR Vβ7.1, Vβ18, and Vβ13.1 monoclonal antibodies, respectively. iNKT cells were analyzed for CD1d PBS57 tetramer reactivity. The unstained control is represented in gray.

Supplementary Figure 3 Statistical analysis of CD1b-restricted lipid antigen presentation by Mo-DCs. **(A)** Mo-DCs from Fabry, Gaucher, NPC, and MPS-VI disease patients and control subjects were loaded with 5 μg/mL of GM1 and co-cultured with the CD1b-restricted T cell clone GG33A. **(B)** Mo-DCs from Fabry and NPC disease patients and control subjects were loaded with 1 μg/mL or 5 μg/mL of sulfatide and co-cultured with the CD1b-restricted T cell clone DS1C9b. The graphs on the left correspond to the cytokine production values. The graphs on the right correspond to the normalized values. Normalization was done for each independent assay considering the highest cytokine production value as 100. Patients are represented with filled symbols and control subjects with open symbols. Each symbol represents the mean of duplicates for the same condition. An unpaired *t*-test (normal distribution) or a Mann-Whitney test (non-normal distribution) was used to compare both groups. *P*-values lower than 0.05 were considered significant. ^*^*p* ≤ 0.05, ^**^*p* ≤ 0.01. The graphs with no symbols relative to statistical analysis means that there were no statistically significant differences.

Supplementary Figure 4 Lipid accumulation in *in vitro* cell models of Fabry and Gaucher diseases. **(A)** Fabry disease *in vitro* cell model: C1R cells treated with DGJ 1 mM, DGJ 1 mM + Gb3:BSA or untreated cells were lysed by sonication. Lipids were extracted and fractioned. The neutral fraction was analyzed by TLC and the Gb3 band intensity was quantified and divided by Sph/Gb4 intensity. **(B)** Gaucher disease *in vitro* cell model: C1R cells treated with CBE 1 mM or untreated cells were lysed by sonication. Lipids were extracted, fractioned and the neutral fraction was analyzed by TLC. GlcCer band intensity was quantified and divided by Sph/Gb4 intensity. GlcCer, glucosylceramide; PE, phosphatidylethanolamine; LacCer, lactosylceramide; Gb3, globotriaosylceramide; Sph, sphingomyelin; Gb4, Globotetraosylceramide; Cer4, ganglioside GM1.

Supplementary Figure 5 Basal production of GM-CSF by **(A)** Mo-DCs and **(B)** monocytes. Mo-DCs or monocytes from Gaucher (G) disease patients, MPS VI (M) disease patient and control (C) subjects were loaded with 50 ng/mL of α-GalCer (GC) or 300 ng/mL of α-Gal-(1-2)-α-GalCer (GGC) and co-cultured or not with an iNKT cell line. After 40 h, GM-CSF was measured in the culture supernatants. Each bar represents mean ± SD of duplicates for the same condition.

Supplementary Figure 6 Statistical analysis of CD1d-restricted lipid antigen presentation by Mo-DCs. **(A)** Mo-DCs from Fabry, Gaucher, NPC, and MPS-VI disease patients and control subjects were loaded with 50 ng/mL of α-GalCer and co-cultured with the iNKT cell clone JS63. **(B)** Mo-DCs from Fabry and Gaucher disease patients and control subjects were loaded with 10 μg/mL of sulfatide and co-cultured with the type II NKT cell clone s33d. The graphs on the left correspond to the cytokine production values. The graphs on the right correspond to the normalized values. The normalization was done for each independent assay considering the highest cytokine production value as 100. Patients are represented with filled symbols and control subjects with open symbols. Each symbol represents mean of duplicates for the same condition. An unpaired *t*-test (normal distribution) or a Mann-Whitney test (non-normal distribution) was used to compare both groups. *P*-values lower than 0.05 were considered significant. The graphs with no symbols relative to statistical analysis means that there were no statistically significant differences.

Supplementary Figure 7 Statistical analysis of CD1d-restricted lipid antigen presentation by monocytes. Monocytes from Fabry and Gaucher disease patients and control subjects were loaded with 50 ng/mL of α-GalCer or 50 ng/mL of α-Gal-(1-2)-α-GalCer and co-cultured with an iNKT cell line. The graphs on the left correspond to the cytokine production values. The graphs on the right correspond to the normalized values. The normalization was done for each independent assay considering the highest cytokine production value as 100. Patients are represented with filled symbols and control subjects with open symbols. Each symbol represents the mean of duplicates for the same condition. An unpaired *t*-test (normal distribution) or a Mann-Whitney test (non-normal distribution) was used to compare both groups. *P*-values lower than 0.05 were considered significant. ^**^*p* ≤ 0.01. The graphs with no symbols relative to statistical analysis means that there were no statistically significant differences.

Supplementary Table 1 CD1b, CD1d, and CD80 expression^*^ on Mo-DCs from LSD patients.

Supplementary Table 2 CD1d, and CD80 expression^*^ on Monocytes from LSD patients.

## Abbreviations

α-Gal A: α-Galactosidase A
α-GalCer: α-Galactosylceramide
APC: antigen presenting cell
BSA: bovine serum albumin
CBE: conduritol B epoxide
DGJ: deoxinojirimycin
ERT: enzyme replacement therapy
Gb3: globotriaosylceramide
GCase: β-Glucosidase
GlcCer: glucosylceramide
GM-CSF: granulocyte-macrophage colony stimulating factor
iNKT: invariant natural killer T
LSD: lysosomal storage diseases
Mo-DCs: monocyte-derived dendritic cells
NPC: Niemann-Pick type C
PE: phosphatidylethanolamine.
