# Supplementary material for: Lipid Antigen Presentation by CD1b and CD1d in Lysosomal Storage Disease Patients
Source: Front Immunol. 2019 Jun 4;10:1264. doi: 10.3389/fimmu.2019.01264 (PMC6558002; doi:10.3389/fimmu.2019.01264)
Supplement: Supplementary Figure 1 — Frequencies of total CD3+ T cells and their CD4+ and CD8+ subsets in Fabry, Gaucher, NPC and MPS-VI disease patients. Total T cells were identified, in PBMCs or CD14− fractions obtained from Fabry, Gaucher, NPC and MPS-VI disease patients or control subjects, by their expression of CD3. Antibodies against CD4 and CD8 were also used to define positive subsets. Circles represent adults (over 16 years of age) and triangles children (under 16 years of age). Black circles identify adult patients that were not under treatment. All the pediatric patients were receiving treatment. Horizontal line represents the mean of each group studied. Normality of each group was analyzed using the D'Agostino & Pearson normality test. To compare patients with the control population, one-way ANOVA (for data with normal distribution) or Kruskal-Wallis test (data with non-normal distribution) were used. **p ≤ 0.01. [file Data_Sheet_1.pdf]

Supplementary material

Supplementary Table 1. CD1b, CD1d and CD80 expression* on Mo-DCs from LSD patients.

Mo-DCs	**Fabry**	**Gaucher**	**NPC**	**MPS-VI**
**CD1b**	1.60 ± 0.64	1.74 ± 0.65	1.63 ± 1.04	0.86 ± 0.36
**CD1d**	1.91 ± 2.09	0.19 ± 0.17	1.85 ± 1.37	1.01 ± 0.52
**CD80**	0.47 ± 0.40	0.64 ± 0.27	1.07 ± 0.57	1.48 ± 0.94
**N**	9	4	9	9

*Mean Fluorescence intensity (MFI) value of each molecule for each patient was relativized using the MFI of control individuals analyzed the same day. Numbers indicate the mean ± SD of N individuals studied.

Supplementary Table 2. CD1d and CD80 expression* on Monocytes from LSD patients.

Monocytes	**Fabry**	**Gaucher**	
**CD1d**	1.02 ± 0.41	0.80 ± 0.03	1.26 ± 0.18
**CD80**	0.98 ± 0.13	1.03 ± 0.06	0.89 ± 0.01
**N**	6	3	4

*Mean Fluorescence intensity (MFI) value of each molecule for each patient was relativized using the MFI of control individuals analyzed in the same day. Numbers indicate the mean ± SD of N individuals studied.

Suppl. Figure 1

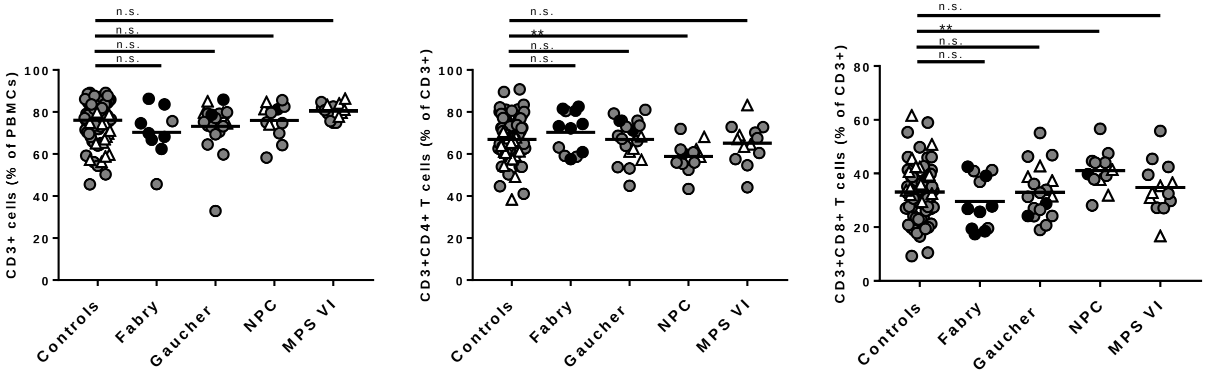

Suppl. Figure 2

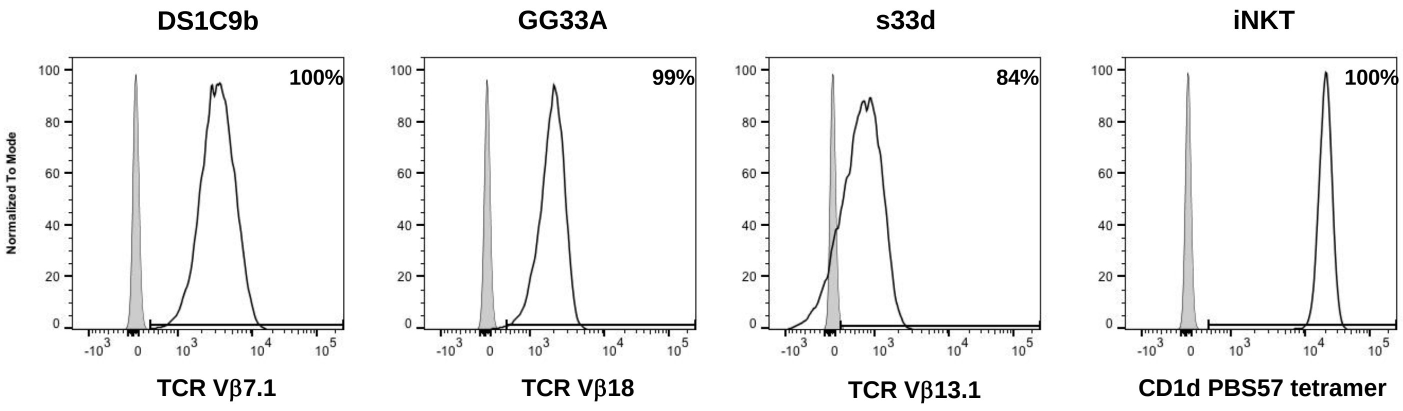

Suppl. Figure 3

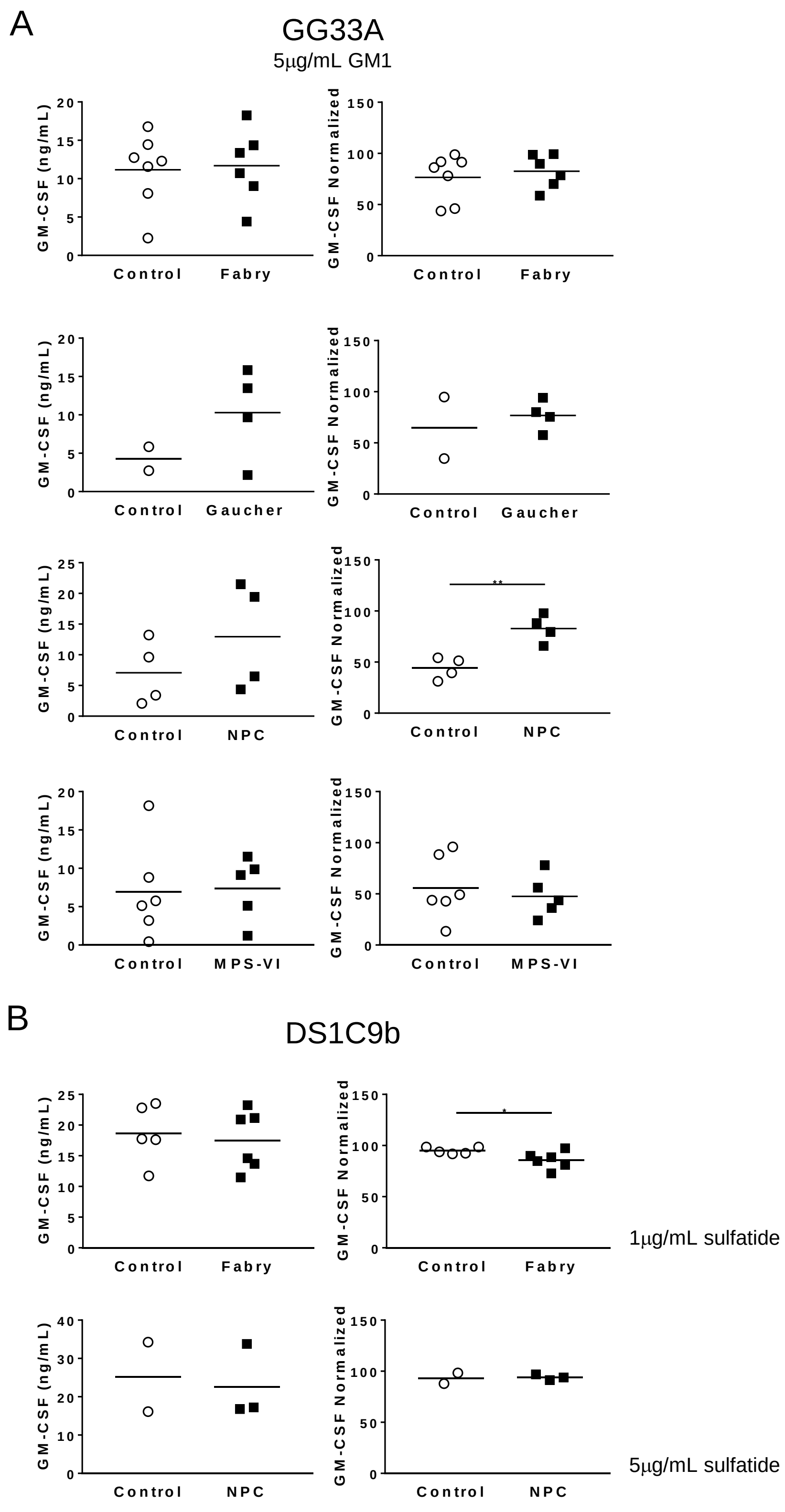

Suppl. Figure 4

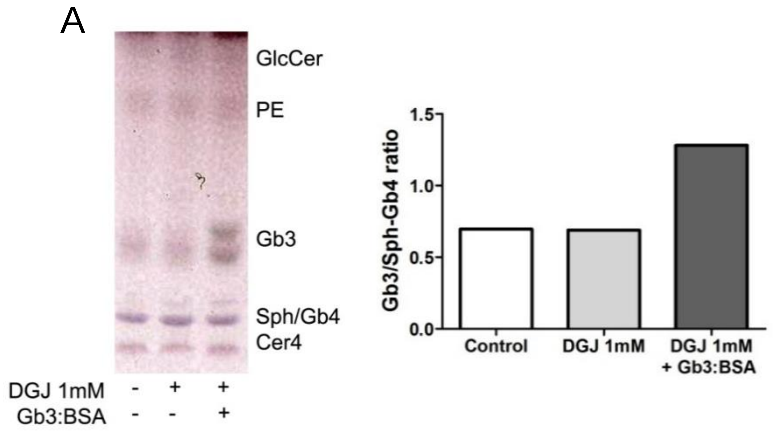

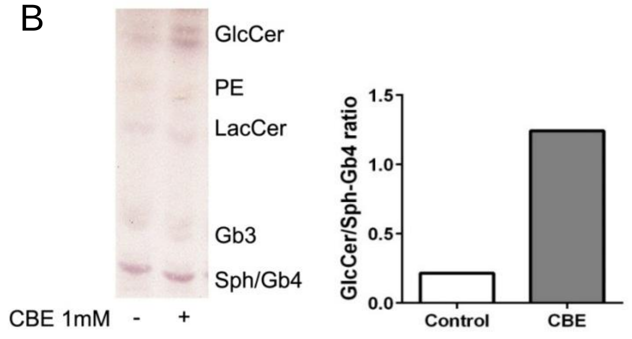

Suppl. Figure 5

A

Mo-DCs

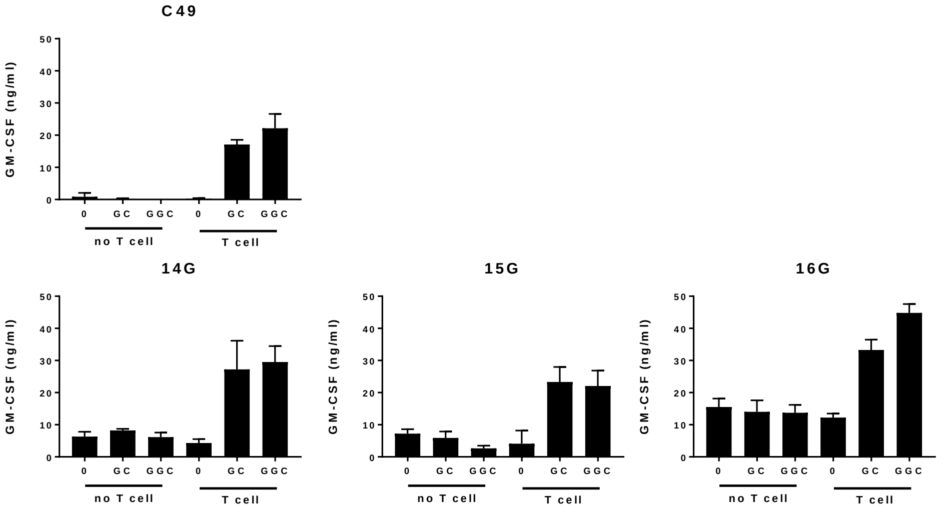

B

Monocytes

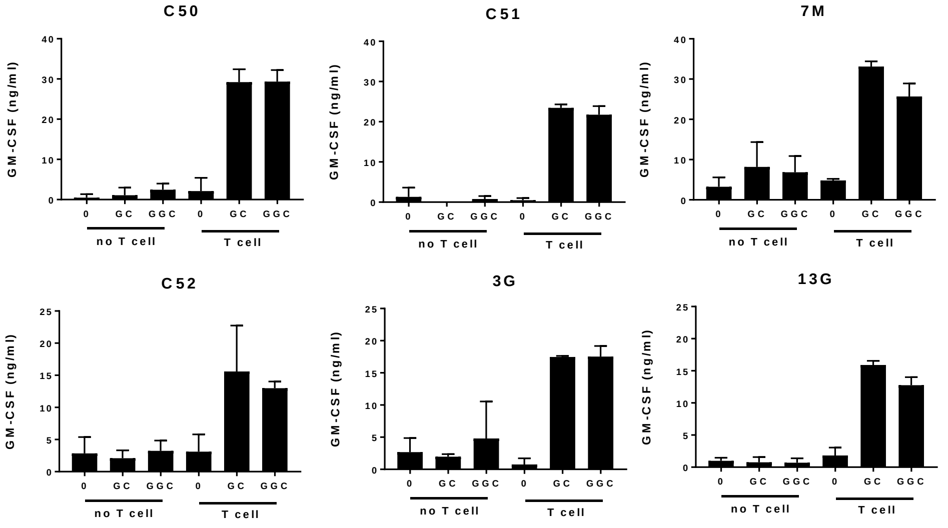

Suppl. Figure 6

A

JS63

50ng/mL α-GalCer

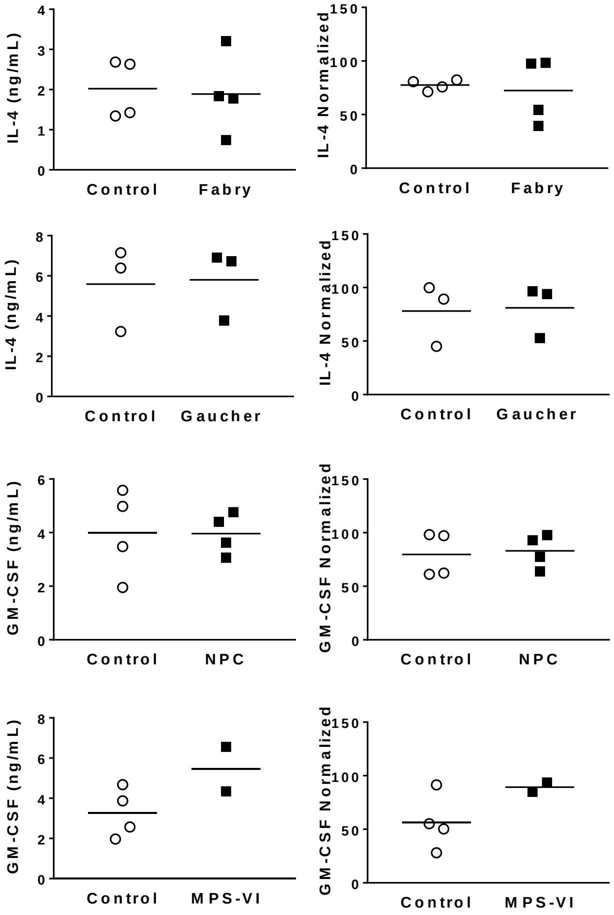

B

s33d

10μg/mL sulfatide

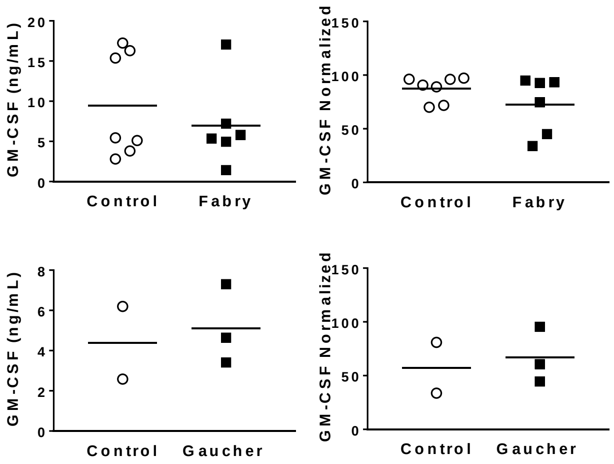

Suppl. Figure 7

iNKT cell line

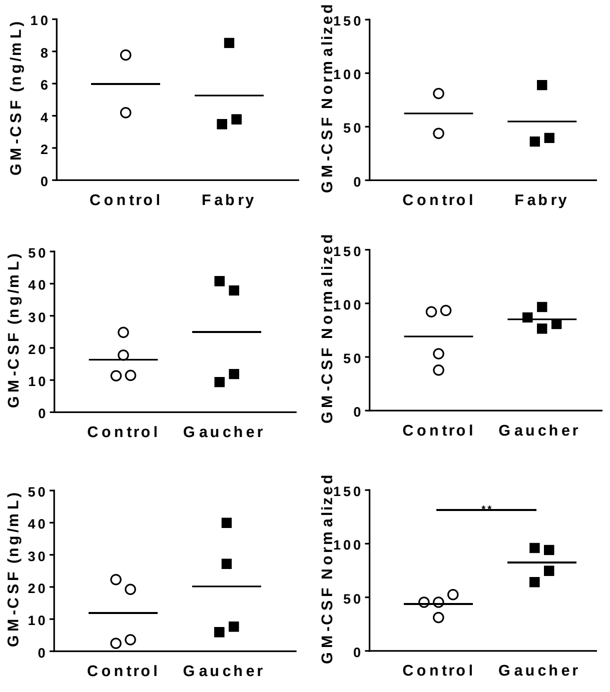

50ng/mL α-GalCer

50ng/mL α-GalCer

50ng/mL α-Gal-(1-2)-α-GalCer
